# Leveraging accreditation to integrate sustainable information literacy instruction into the medical school curriculum

**DOI:** 10.5195/jmla.2018.276

**Published:** 2018-07-01

**Authors:** Natalie Tagge

**Affiliations:** Education Services Librarian, Ginsburg Health Sciences Library, Temple University, Philadelphia, PA

## Abstract

**Background:**

While the term “information literacy” is not often used, the skills associated with that concept are now central to the mission and accreditation process of medical schools. The simultaneous emphasis on critical thinking skills, knowledge acquisition, active learning, and development and acceptance of technology perfectly positions libraries to be central to and integrated into the curriculum.

**Case Presentation:**

This case study discusses how one medical school and health sciences library leveraged accreditation to develop a sustainable and efficient flipped classroom model for teaching information literacy skills to first-year medical students. The model provides first-year medical students with the opportunity to learn information literacy skills, critical thinking skills, and teamwork, and then practice these skills throughout the pre-clerkship years.

**Conclusions:**

The curriculum was deemed a success and will be included in next year’s first-year curriculum. Faculty have reported substantial improvements in the information sources that first-year medical students are using in subsequent clinical reasoning conferences and in other parts of the curriculum. The effectiveness of the curriculum model was assessed using a rubric.

## BACKGROUND

Many changes to medical education in the past decade have been the result of the recommendations of the Association of American Medical Colleges (AAMC) and the Liaison Committee on Medical Education (LCME). These changes have led to the development of a new medical education model that emphasizes “critical thinking, lifelong learning skills” [1]. To help students achieve learning outcomes related to these broad goals, new instructional strategies and technologies can and have been leveraged. One significant and widely adopted example is the flipped classroom model, which frees up in-class time for active learning and formative assessment. The flipped classroom model allows students to be introduced to concepts in prework in the form of readings, video lectures, or activities and “then come together to drive beyond the basics to develop skills that cannot be taught in a lecture” [2]. With this model, it is much easier to provide meaningful interventions and guidance while determining whether students are practicing teamwork and developing knowledge acquisition skills [3].

This model is well aligned to the Association of College & Research Libraries (ACRL) Framework for Information Literacy for Higher Education frames, particularly “Searching as Strategic Exploration” [4]. Furthermore, the case-based format commonly used during the in-class portion of a flipped classroom model allows information literacy skills to be introduced naturally and pragmatically to students [5].

In 2015, LCME recommended that the Temple University Lewis Katz School of Medicine integrate more active learning into the curriculum. LCME also discussed the program objective “self-directed and life-long learning” [6], which is philosophically similar to information literacy and includes the goals of independent identification and critical appraisal of information.

In 2016, the School of Medicine began integrating more active learning methods into the curriculum through clinical reasoning conferences (CRCs). These conferences were designed in collaboration with the Temple University Center for Advancement in Teaching to address medical students’ self-directed and lifelong learning skills. The CRCs follow a flipped model in which students are given a clinical case, pre-conference materials (typically readings), and an evaluative test prior to the in-person conference. During the conference, students work in groups based on their doctoring college, a four-year academic community in which students learn clinical skills. Students are given the clinical case questions, are asked to find the answers, and present their results to the class.

During CRCs, students need basic information literacy skills so that when they are confronted with a question, they can think critically about appropriate places to search for the information that is needed to answer the question and evaluate the appropriateness and accuracy of the information for their needs. The School of Medicine found that many students were citing dubious sources and were not able to successfully evaluate or analyze their source selection, leading faculty to reach out to the Temple University Ginsburg Health Sciences Library for assistance.

## STUDY PURPOSE

This case study discusses how one health sciences library collaborated with a medical school to integrate information literacy skill building into a flipped classroom curriculum model that provides students with the opportunity to learn information literacy skills, critical thinking skills, and teamwork and then practice these skills throughout their pre-clerkship years. The effectiveness of the curriculum model was assessed using a rubric (supplemental Appendix A).

## CASE PRESENTATION

During a series of meetings in spring 2016, a group of medical faculty (including a dean) and librarians (including the library director) met to discuss medical students’ information literacy needs and ways that the library could help address these needs. In the first meeting, librarians listened to the medical faculty’s concerns and the issues that they had observed in the CRCs. The group came to the consensus that the best way to get students to concentrate on thinking about information was to design one library-led CRC for first-year medical students that focused more on process than answers. A medical faculty member and librarian would work collaboratively to create this CRC. The CRC would consist of librarian-created videos to be watched prior to the CRC, a quiz that tested the students’ learning from the videos, and faculty-written cases and case questions.

The group began the next conversation with librarian-written learning outcomes that were linked to both the ACRL Framework for Information Literacy in Higher Education and the LCME competencies, curricular objectives, and curricular design (Table 1) [4, 6]. This gave the faculty and librarians a foundation to ensure that they all agreed on the goals of the CRC and that every instructional decision made in designing the CRC was connected to the agreed-upon learning outcomes. A series of six interactive videos were scripted, recorded, and edited using Camtasia. The videos are available from the Temple Health Sciences Libraries website (interactive multiple-choice questions were removed to meet Temple Libraries Americans with Disabilities Act Compliance Guidelines).

**Table 1:** Librarian-written learning outcomes mapped to the ACRL Framework for Information Literacy in Higher Education and the LCME competencies, curricular objectives, and curricular design

Library-led clinical reasoning conferences (CRCs) learning outcome	Mapped to Association of College & Research Libraries (ACRL) Framework	Mapped to Liaison Committee on Medical Education (LCME) Competencies
1. First-year medical students will recognize when they need outside information.	Searching as strategic exploration	Self-directed learning involves medical students’ self-assessment of learning needs
2. First-year medical students will match their information needs and search strategies to appropriate search tools.	Searching as strategic exploration	Independent identification…of relevant information
3. First-year medical students will be able to evaluate information to determine if the information is appropriate for their needs.	Authority is constructed and contextual	Appraisal of the credibility of information sources

The video topics ranged from a basic introduction to the library website, to a discussion of moving from questions to searches, to a discussion of evaluating health sciences information. The videos were directly mapped to the learning outcomes (Table 2). A six-question multiple choice pretest was administered via the medical school’s exam software to test student learning from the videos (supplemental Appendix B). Students accessed the videos and the link to the quiz via the medical school’s course management system.

**Table 2:** Videos mapped to learning outcomes*

Video	Learning outcomes
Ginsburg Library website tour (4:10 minutes)	2
Answering a clinical question with a textbook or point-of-care tool (6:19 minutes)	1, 2, and 3
Answering a clinical question with a research or review article (9:11 minutes)	1, 2, and 3
DynaMed search demonstration (3:11 minutes)	2
PubMed search demonstration (4:33 minutes)	2
Evaluating health information (3:32 minutes)	3

* As listed in Table 1.

In tandem, a faculty member developed a series of three clinical cases with four or five questions per case. The faculty member drafted learning outcomes with librarian input for the cases and questions (Table 3). A librarian collaborated with the faculty member to develop a faculty answer guide for use during the CRC that provided suggested resources and search terms for each case question. Both faculty and librarians had access to the guide during the CRC so that they could provide students with consistent advice.

**Table 3:** Medical faculty member–written clinical case learning outcomes

Clinical case learning outcomes
To learn how to use the Ginsburg Health Sciences Library website and resources to search for information
To locate, recognize, and utilize the essential types of literature resources (textbook, point-of-care, review article, research article)
To translate a question into searchable language (search terms, filters, etc.)
To begin to think critically about information sources

During the CRC, 4 librarians and medical school faculty facilitated the 2-hour, back-to-back sessions as 200 students (100 per session) worked in groups of 6–8 to answer the clinical case questions by consulting external information sources. For example, one case described a 64-year-old woman with symptoms that students were expected to diagnose as venous thromboembolism (VTE). One question associated with this case was, “What does the current research indicate about the predictors of VTE and the time frame of VTE in breast cancer patients? Show your search and cite your sources.” Students were required to bring a laptop to the session so that they could search for the answers to the clinical case questions and document their search processes. For each case, a randomly selected student group presented their answers to the clinical case questions and their search processes, and were given verbal and written feedback. The written feedback form was standardized and used in all CRCs, including the library-led CRC. The form was developed by the medical school and gives feedback in the areas of presentation skills, content, professionalism, and teamwork.

The library-led CRC was repeated in fall 2017 with minor changes. One case and its respective questions were removed to allow students more time to focus on their search processes. In fall 2017, the library also had an opportunity to conduct a rubric assessment of the CRC. The rubric “Information Literacy in Student Work Rubric—Temple Health Sciences Libraries” (hereafter referred to as the Temple HSL Rubric) was adapted from the Claremont Colleges Library Information Literacy in Student Work Rubric (supplemental Appendix A) [7]. The Temple HSL Rubric assesses inquiry, evaluation of evidence, and communication of evidence as exhibited in the student CRC presentations. It includes 4 evaluation levels (1=initial, 2=emerging, 3=developed, and 4=highly developed).

Two librarians assessed both sessions, and a third librarian assessed the second session. The three librarians used the rubric to score student (i.e., presenter) answers to each case question. A mean for the three rubric scores (inquiry, evaluation, and communication) across questions was compiled for each case in each session. Finally, the mean and standard deviation for each area rated (inquiry, evaluation, and communication) was computed for the cases across sessions and raters (Table 4). Students scored in the “developed” level for all three rated areas—”Inquiry,” “Evaluation,” and “Communication”—for the two cases used in fall 2017.

**Table 4:** Mean and standard deviation, Temple HSL rubric assessment, three raters

Case	Inquiry		Evaluation		Communication	

	M	SD	M	SD	M	SD
1	3.47	(0.57)	3.49	(0.39)	3.40	(0.42)
2	3.18	(0.46)	3.22	(0.40)	3.31	(0.31)

M=mean.

SD=standard deviation.

## DISCUSSION

The library-led CRC will be included again in the 2018 first-year medical curriculum. Faculty have reported substantial improvements in the information sources that first-year medical students have used in subsequent CRCs and other parts of the curriculum. They have also reported that first-year medical students are using much better information sources than medical students who did not receive this instruction. While anecdotal data from faculty and students is important to the success and continuation of this collaboration, formal assessment such as the rubric assessment of the CRC conducted by the library is necessary [8]. Students’ scores at the “developed” level indicate that there is still room for student skill improvement but also indicate that the library-led CRC materials including the videos, cases, and questions are not too advanced for first-year medical students.

The flipped classroom design of the CRC allowed librarians to provide students with important information, such as the basics of searching PubMed, in prework and then focus on assisting students with their search process in the workshop [9]. This allows students to engage in authentic learning and librarians and faculty to facilitate the questions and issues that students encounter [10]. In our case, medical school faculty also learned from librarians. For example, multiple faculty members said that they did not know about PubMed search details, and this newfound knowledge improved their ability to retrieve relevant results.

Furthermore, librarians had the opportunity to learn how faculty approached finding evidence in clinical scenarios [8]. In one particularly enlightening moment, a faculty member shared with the students that he sometimes excuses himself from a patient’s room by saying he is going to wash his hands but instead will perform a quick search to verify a diagnosis. It would be unlikely that students or librarians would ever have this intimate knowledge in a guest lecture–structured library instruction session.

The library-led CRC has limitations and unique challenges. First, because the workshop was guided by the students’ challenges and example search processes, some librarians who were involved were concerned that some topics, including Medical Subject Headings (MeSH), were not sufficiently explained. Second, librarians who were used to providing rehearsed guest lectures found it challenging to improvise answers to questions. Third, while collaborating with the medical school and integrating into the curriculum were positive experiences, librarians had to give up some control. For example, a librarian wrote a pre-test question that asked students to perform a search in PubMed. However, students were unable to perform the search because access to other computer programs was locked down by the medical school exam software.

The curriculum-integrated CRC design allowed librarians to work as content experts collaborating with medical faculty. Medical students had an opportunity to engage with medical faculty and librarians, each providing unique expertise to the search process. The medical school now views the library as a curriculum partner [11]. The library is currently in discussion to collaborate on an information literacy–focused CRC for second-year medical students. An ideal outcome would be a program that scaffolds skills across all four years of medical school [12]. The increasing importance of the “Core Entrustable Professional Activities for Entering into the Residency” in medical education has the potential to further solidify the library as a curriculum partner [13, 14]. The model also has the potential to be translated into other health sciences programs that the library serves, such as the dental school [15].

## SUPPLEMENTAL FILES

Appendix A Information literacy in student work rubric, Temple Health Sciences Libraries (version 2017/18)

Appendix B Clinical reasoning conferences pretest questions
