# Supplementary material for: Leveraging accreditation to integrate sustainable information literacy instruction into the medical school curriculum
Source: J Med Libr Assoc. 2018 Jul 1;106(3):377–82. doi: 10.5195/jmla.2018.276 (PMC6013141; doi:10.5195/jmla.2018.276)
Supplement: Appendix A [file jmla-106-377-s001.pdf]

JMLA Journal of the Medical Library Association

SC
Supplemental content to
J Med Libr Assoc. Jul;106(3):dx.doi.org/10.5195/jmla.2018.276
www.jmla.mlanet.org
© Tagge 2018

Leveraging accreditation to integrate sustainable information literacy instruction into the medical school curriculum

Natalie Tagge, MS

APPENDIX A

Information literacy in student work rubric, Temple Health Sciences Libraries (version 2017/18)

Learning outcome	Level of achievement			
	Highly developed<br>4	Developed<br>3	Emerging<br>2	Initial<br>1
**Inquiry**	*Expert articulation of information need.*<br>• Constructs highly effective research strategy (e.g., keywords, sources) demonstrating sophisticated thought	*Satisfactory articulation of information need.*<br>• Research strategy contains some flaws (e.g., misses obvious keyword synonyms or major databases) but is sound overall	*Partial articulation of information need.*<br>• Research strategy contains multiple or major errors (e.g., irrelevant keywords or sources)	*Poor to no articulation of information need.*<br>• Poor to no research strategy (e.g., no keywords or sources)
**Evaluation of evidence**	*Source materials employed demonstrate expertise and sophisticated independent thought.*<br>• Uses appropriate and authoritative sources to support claims<br>• Demonstrates a knowledge of evidence and sources selected	*Source materials are adequate and appropriate but lack variety or depth.*<br>• Sources are used to support claim(s) but might not be the most authoritative source to make claim<br>• Demonstrates a preliminary critical exploration and knowledge of evidence, and sources selected	*Source materials used are inadequate.*<br>• Relies on largely inappropriate sources<br>• Clearly selected sources out of convenience<br>• Demonstrates little critical exploration and knowledge of sources selected	*Source materials are absent or do not contribute to claim(s) or argument(s).*<br>• When included, sources are too few or badly inappropriate<br>• No evidence of critical exploration and knowledge of sources selected
**Communication of evidence**	*Evidence is integrated and synthesized expertly to support claims.*<br>• Consistently presents evidence to support claim(s) and argument(s)<br>• Synthesizes and contextualizes evidence appropriately for audience	*Proficient synthesis and integration of evidence.*<br>• Generally employs evidence to support claim(s) and argument(s)<br>• May present some evidence without context	*Weak attempts at synthesis or integration.*<br>• Sporadically uses evidence to support claim(s) or argument(s)<br>• Frequently fails to put sources into context (e.g. "says...")	*No evidence of attempt at synthesis or integration.*<br>• Claim(s) or argument(s) lack necessary evidence<br>• Fails to contextualize evidence

JMLA Journal of the Medical Library Association

SC
Supplemental content to
J Med Libr Assoc. Jul;106(3):dx.doi.org/10.5195/jmla.2018.276
www.jmla.mlanet.org
© Tagge 2018

Identification

Group # ____________________ Scorer name ____________________ Program/Year____________________

Quality of attribution, evaluation, and communication of IL (see rubric for details):

	Highly developed (4)	Developed (3)	Emerging (2)	Initial (1)	Comments	Totals
**Inquiry**						
**Evaluation of sources**						
**Communication of evidence**						
					Sum:	

Edited 2017 by Natalie Tagge for the Temple Health Sciences Libraries. Rubric content adapted for the Claremont Colleges by Char Booth (char_booth@cuc.claremont.edu), Sara Lowe (sara_lowe@cuc.claremont.edu), Natalie Tagge (natalie_tagge@cuc.claremont.edu), and Sean Stone (sean_stone@uc.claremont.edu) from an instrument originally developed at Carleton College, Gould Library Reference and Instruction Department. Information literacy in student writing rubric and codebook. Northfield, MN: Carleton College; 2012. <http://go.carleton.edu/6a>). *This rubric version (2017/18) was revised summer–fall of 2017 and finalized September 2017.*
