# Supplementary material for: Leveraging accreditation to integrate sustainable information literacy instruction into the medical school curriculum
Source: J Med Libr Assoc. 2018 Jul 1;106(3):377–82. doi: 10.5195/jmla.2018.276 (PMC6013141; doi:10.5195/jmla.2018.276)
Supplement: Appendix B [file jmla-106-377-s002.pdf]

JMLA Journal of the Medical Library Association

SC

Supplemental content to
J Med Libr Assoc. Jul;106(3):dx.doi.org/10.5195/jmla.2018.276
www.jmla.mlanet.org
© Tagge 2018

Leveraging accreditation to integrate sustainable information literacy instruction into the medical school curriculum

Natalie Tagge, MS

APPENDIX B

Clinical reasoning conferences pretest questions

Q1. What is the best search to use in DynaMed to find the answer to this question: What are the symptoms of splenic injury?

A. Splenic injury
B. What are the symptoms of splenic injury?
C. Splenic injury AND symptoms

Correct Answer: Splenic injury

Q2. Do a search for bee allergy in DynaMed? What is a **possible** risk factor?

A. Spending time outdoors
B. Bee keeping
C. Consuming grape juice or newly pressed wine

Correct Answer: Consuming grape juice or newly pressed wine

Q3. What is the goal of a systematic review article?

A. Answer a focused clinical question
B. Provide a summary or overview of a topic
C. Report on original research

Correct Answer: Answer a focused clinical question

Q4. What is a good method of finding a review article about lung cancer in PubMed?

A. PubMed does not contain review articles
B. Search for lung cancer and use the filters to limit to Review
C. Search for lung cancer AND review article

Correct Answer: Search for lung cancer and use the filters to limit to Review

Q5. Do a search for heart attack in PubMed. According to the search details, what is the Medical Subject Heading (MeSH) for heart attack?

A. Myocardial Infarction
B. Heart Attack
C. There is no MeSH for heart attack

Correct Answer: Myocardial Infarction

Q6. What is one advantage of textbooks being available in an online format?

A. Online textbooks are free
B. Online textbooks are easy to search
C. Both A and B

Correct Answer: Online textbooks are easy to search
